# Synthesis of nanogate structure in GO-ZnS sandwich material

**DOI:** 10.1038/s41598-018-37396-8

**Published:** 2019-01-30

**Authors:** Praveen Kumar, Meitram Niraj Luwang

**Affiliations:** 1 Chemical Engineering and Process Development Division, National Chemical Laboratory, Pune, 411008 India; 2 Academy of Scientific and Innovative Research (AcSIR), CSIR-Human Resource Development Centre, Campus Postal Staff College Area, Ghaziabad, 201002 India

## Abstract

Graphite Oxide (multi-layer) composite with other materials has a huge application in various field of science, due to its excellent and unique properties. Even though from past decade, immense research has been done by materials scientists in this field, but the chemistry is still not yet satisfactory. Here, in this work, through the discovery of Nanogate structure, we have reported for the first time the experimental results that enlightened the clear chemistry between the GO and ZnS which is further supported by the DFT calculations. This novel synthesis method led to the discovery of nanogate structure sandwiched between the GO layers. The nanogate formation also shows enhanced properties for various applications like photocatalytic activities, etc. Due to the nanogate formation, there might be a possibility of enormous generation of electrons on excitation of the composite materials, which can be a boom for various applications like photocatalysis, water splitting, solar cell, etc.

## Introduction

Graphite Oxide (GO) is a sp^2^ hybridized layered hexagonal arranged carbon materials^1,2^ which originated by the functionalization of graphite by the well-known modified Hummer method^3^. The different functional group are attached at basal plane as well as the edge side of graphite sheet, or in detail, the –OH and epoxy group are attached at basal plane while –C=O and carboxylic group are attached on the edge of the sheet^4^. Due to this functionalization, it is easily dispersed in water as these functional group increases the hydrophilic nature of graphite sheet^5^. GO have garnered a huge importance in various field like catalysis^6^, electronic^7,8^ and other biomedical applications^9^. When graphite oxide (GO) is converted into reduce graphite oxide (rGO)^10^, its properties totally changes. The reduction process can be achieved by various categories such as thermal^11,12^, chemical^10,13^ and electrical method^14^. Totally reduced graphite oxide is called graphene^15^ but complete reduction is impossible, that means partial reduction takes place or some of functional groups are reduced and other are eliminated or changed into other functional group during the reduction procedure^16^.

Researchers have reported on the enhancement of the properties of graphite oxide by the variation of the composition and doping/incorporation with other materials^17,18^. Incorporation of quantum dots into GO sheet is on boom from past more than half decade^19^. Indrajit *et al*.^20^ have reported that metal nanoparticles incorporated in to graphene oxide heterogeneous catalyst enhances the photocatalytic activity. As GO is a partially insulating candidate with wide band gap, but when it reduced by other materials, it shows a great change in its property like photo catalytic activity^21^ water splitting^22^, hydrogen evolution^23^, sensing^24,25^ and many other electronic based applications^26,27^. Recently, Z. Wang *et al*.^28^ demonstrated that the electritical conductivity of rGO can be enhanced by doping it with thiophene-S.

Although graphene and its derivatives plays a vast role in chemistry, physics and biological field^29^ but the indepth chemistry is not completely understood. That is the reason why, from the past decade it has become a hot topic of research and many research groups are trying to know the chemistry behind which led to a huge variation in its properties with different functional group. Very recently Pumera *et al*.^30^ have reported an allylic C-H bond functionalization of hydrogenated graphene through a dehydrogenative cross-coupling reaction. As mentioned above, GO has four type of functional group on its surface and their chemistry has been studied extensively but still there is a lack of clear understanding of its chemistry. Out of all the functional group reported in GO, epoxide groups are the most reactive species which makes the GO surface very sensitive^31^, leading to the variation of its properties when it forms composite with other materials^32^. Due to the three member epoxide ring strained, it can be easily opened^33^, as also reported in one of the studies where the epoxide ring opening is successfully done through the nucleophilic attack of sulphur^34^. The new understanding behind the epoxide ring opening through other compound leads to formation of new structures on the reduced GO layer, thereby opening a new window for an attractive field of chemistry research.

Recently, Dev *et al*.^35^ have reported a theoretical work on stabilizing graphene-based organometallic sandwich structure through defect engineering. Similar work has also been reported by Shahsavari *et al*.^36^ where they carry out the first theoretical study of the electronic and optical properties of 2D metal oxide sandwiched between the bilayer of graphene. Both the work can enlighten on the formation of sandwich structure of graphene with any molecule theoretically but they also claim the difficulty and lack of experimental data in the similar field.

Herein, for the first time we are reporting an experimental as well as theoretical result showing the successful incorporation of a new chemical bond entity in between the GO layers. The incorporation was successful due to the novel synthesis methodology and the new chemical bond entity act as a gate for the movement of the electrons in between the layers and it has been named as “Nanogates” due to its gate like structure and properties.

## Understanding the “Nanogates”: Reaction Mechanism

**Figure 1:**
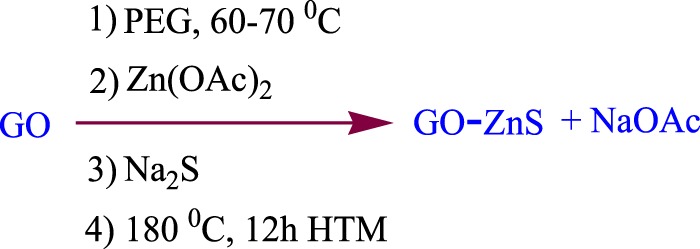
Reaction scheme for the formation of GO-ZnS sandwich structure.

The Nanogate moiety was obtain by the reaction of GO and ZnS through the following novel reaction conditions (Fig. 1). The novelty of this synthesis method is the incorporation of the spacer (Zn acetate - PEG) in between the GO layers which increase the interlayer distance, thereby allowing the reactant to enter and react with the interlayer functional groups. This has led to the formation of the nanogate structure in the GO-ZnS sandwich material. Detailed synthesis procedures are presented in Section S1.1 (See Supplementary Information).

**Figure 2:**
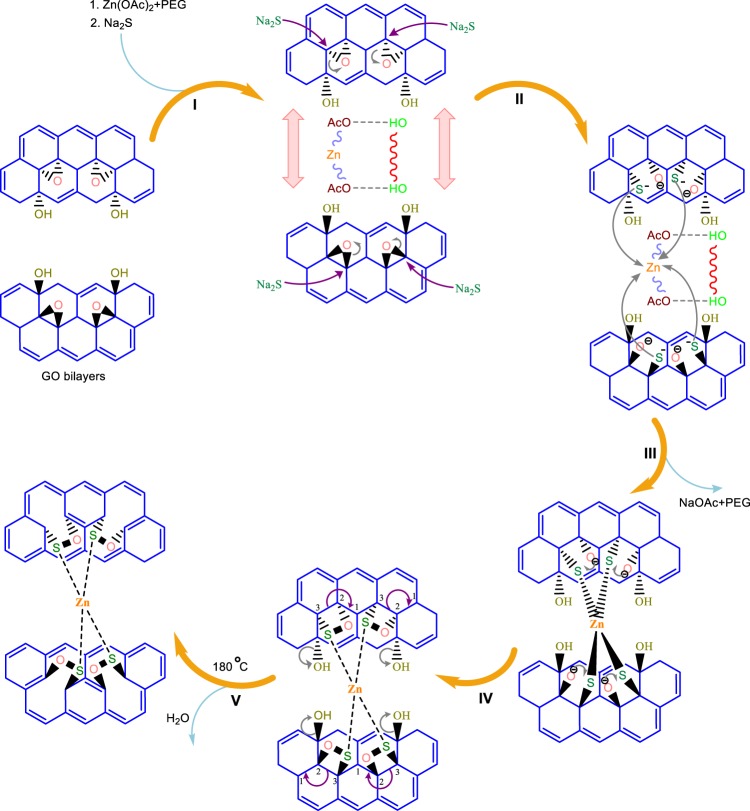
Proposed reaction mechanism for the formation of GO-ZnS sandwich structure.

The reaction scheme can be further explained in detail by the proposed reaction mechanism as shown in Fig. 2. Initially (Step I), the interlayer distance between the two GO sheets is widening due to the incorporation of the Zn acetate - PEG moiety. PEG and Zn acetate link together through H-bonding and due to the presence of many O-atom, it is negatively charged, which further repel the 2 GO layer which is also impregnated with many O-atoms. Due to the widening of the layers, it is easier for the nucleophile (Na2S) to attack the epoxide ring (Step II). After the epoxide ring opening, the negatively charged S-ion is able to link with the Zn-ion through a covalent bond (Step III), as zinc ion is positively charged, it can easily withdraw the electron from 3p orbital of S atom in its own 4p orbital (Ligand to Metal Charge Transfer; LMCT). Here, Zn get partial negative charged and S get partial positive charged. Due to the partial positive charge on S atom through LMCT, it can bond with O-atom (negatively charged), resulting in a four member ring closing (Step IV) thereby converting the covalent bond between Zn and S into coordination bond. This process is called Metal to Ligand Charge Transfer: MLCT (discussed later). Due to hydrothermal treatment at 180 °C, there is a 1–3 hydride shift (bond breaking) in the GO layer leading to the defect formation (Line defect) followed by the dehydration (Step V). This further leads to the conjugation in the GO layer making it an overall stable material with nanogates structure^37,38^.

To confirm the proposed reaction mechanism and the nanogate structure, different characterisation techniques has been performed. (See Supplementary Information Section S1.2)

## Indepth Analysis of the “Nanogate” Structure

**Figure 3:**
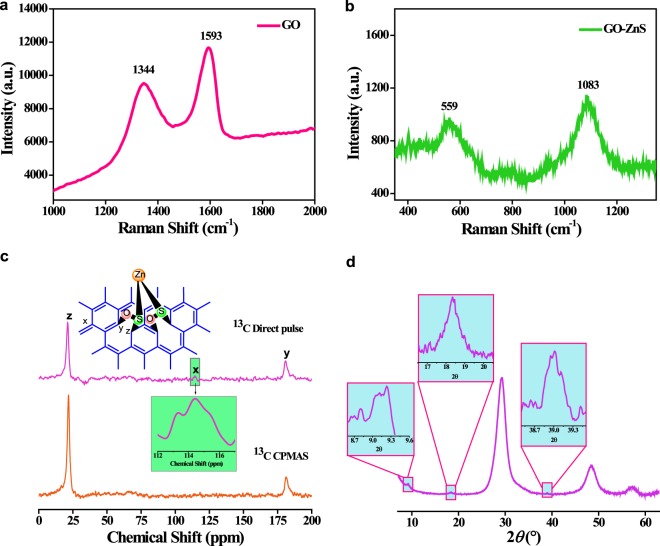
Different types of characterisation technique performed during the confirmations of GO-ZnS sandwich structure. (**a**,**b**) are the Raman spectra of GO and sandwich material (**c**), solid state CP- MAS NMR were measured at an external field B_0_ 9.38977 T using [400 MHz] JEOL 400 spectrometer operating at ^13^C larmor frequency of 100.5253 MHz. Cross-polarisation magic angle spinning (CP-MAS) were recorded with 1s recycle time, and 1 ms contact time, 67,000 scans and 10,000 scans for direct pulse. The ^13^C chemical shift were calibrated against TMS (δ = 0 ppm). ^13^C CP-MAS and direct pulse spectra of sandwich material, (**d**) wide angle X ray diffraction (WXRD) pattern.

Raman spectroscopy is one of the powerful tools to extract the information of new chemical functionalities in a compound. Figure 3a,b shows the Raman spectra of the synthesised GO and GO-ZnS sandwich material. Raman shifts are observed at 1344 cm^−1^ and 1593 cm^−1^for GO which corresponds to D and G band whereas in GO-ZnS the peak at 560 cm^−1^ and 1084 cm^−1^ indicating the new chemical bond of C-S bond and S-O bond respectively^39^. This new peak of C-S and S-O has been observed for the first time in GO-ZnS sandwich materials, till date. Further confirmation of the new bond formation has been done from the FTIR analysis.

NMR analysis has been performed for further confirming and elucidating the exact structure of GO-ZnS sandwich structure. Figure 3c shows the ^13^C SS-NMR spectra of GO in solid state where three major peak at 61 ppm, 71 ppm, and 131 ppm (see Supplementary Information, Section S2.1) corresponds to the epoxide, hydroxyl and sp^2^ cluster and other minor peaks at 168 ppm and 191 ppm corresponding to carbonyl and carboxylic group, respectively. All data are perfectly matched with already reported chemical shift of GO^40^. In synthesized GO-ZnS sandwich materials, major peaks observed at 21 ppm corresponds to sp^3^ carbon attach to sulphur while 181 ppm to the sp^2^ carbon attach to oxygen on GO plane. It verifies the new bonding between hybrid atoms (S and O) to adjacent carbon atom (after epoxide ring opening) attached to the basal plane of GO, as already reported in literature^41–43^. As ^13^C direct pulse experiment gives the more detailed confirmation than ^13^C CPMAS, Fig. 3c direct pulse spectra shows one more signal around at 114.4ppm which corresponds to the sp^2^ carbon in sandwich material, which is not observed in ^13^C CPMAS spectra. To the best of our knowledge chemical shift (δ) at 21 ppm analogous to C-S bonding and (δ) value at 181 ppm corresponding to C-O bond. NMR analysis confirms the formation of new C-S and S-O bond in the new sandwich material.

**Figure 4:**
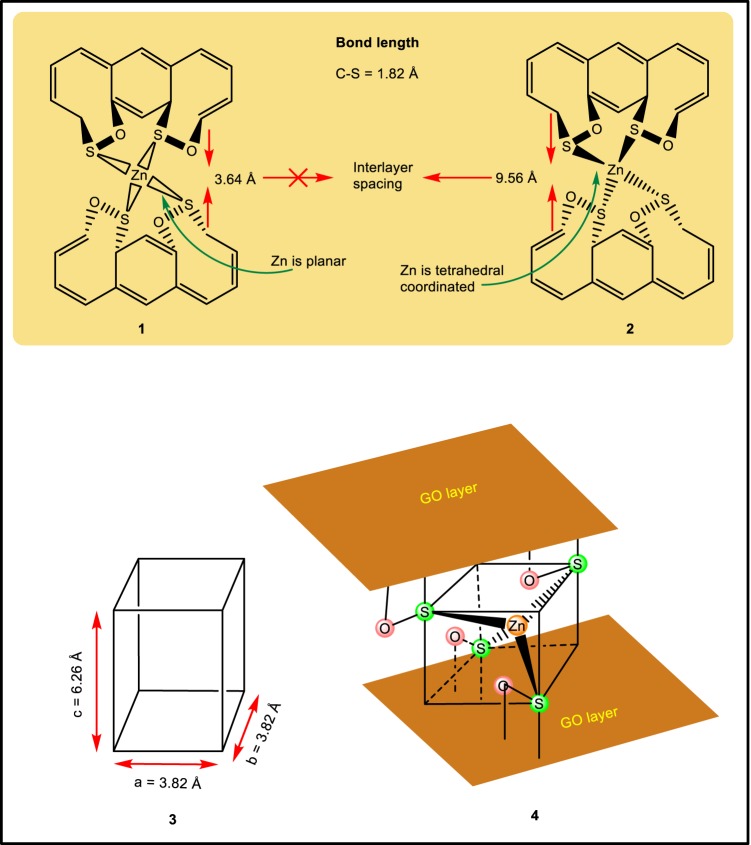
Justification of tetrahedral coordinated Zn through 4-S atom between GO layers. Pictorial 1 when Zn is planar then the interlayer space between layers is around 3.64 Å which doesn’t match with experimental observed value (9.56 Å). Pictorial 2 when Zn is tetragonally corordinated with 4 S-atom, the calculated interlayer distance is 9.9 Å which is very close to the experimental value. Pictorial 3 Primitive unit cell for wurtzite structure for [ZnS_4_], Pictorial 4 shows the tetrahedral coordinated ZnS unit cell between two GO layers in sandwich material.

Wide angle XRD analysis as shown in Fig. 3d further confirms the formation of the new chemical bond entity (nanogates) between two GO layers. The peak positions of GO are shifted towards the lower 2θ values while that of ZnS shows no observable changes (see Supplementary Information, Section S2.2). It is a well-known fact that a decrease in the 2θ values indicates the increase in the d-spacing of a compound which confirms that the interlayer spacing between the two GO sheets has been increased due to the incorporation of ZnS moiety leading to the formation of the proposed hetero nanogates structure. To actually confirm the above point, the interlayer distance between the two GO sheets has been measured and it is found to be 9.56 Å (001). As shown in Fig. 4, when the ZnS moiety is consider to be in a planar form, the overall distance between the two GO sheets is found to be 3.64 Å (C-S bond distance is 1.82 Å). But when the Zn atom is tetrahedrally coordinated with the S atom, it gives a unit cell distance of 6.26 Å^44^. So, along with the two C-S bond (nanogates) on both the upper and lower layers, it gives an overall total distance of 9.9 Å which is very much closed to the experimental value. The difference of 0.3 Å can be assigned due to the slight variation in the bond length of Zn-S (coordination bond) in the GO-ZnS sandwich materials. Generally under normal condition, the most stable form of ZnS is wurtzite structure in which Zn is bonded with four S atoms [ZnS_4_] in a tetrahedral fashion.

**Figure 5:**
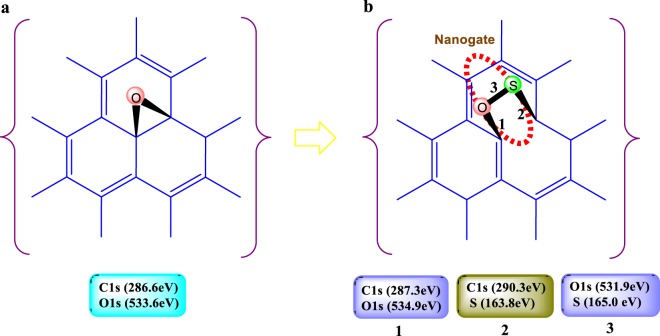
Discovery of hetero nanogate structure and confirmation of S-O and C-S bond in the GO-ZnS sandwich material on the bases of XPS data. (**a**), binding energy of epoxide group (C-O-C) are 286.6 eV and 533.6 eV corresponding to C1s and O1s spectra of GO (see supplementary information). (**b**), 1 denote the C-O bond with red sphere O bond shows its binding energy 287.3 eV and 531.9 eV corresponding to C1s and O1s spectra respectively, 2 indicate the C-S bond with green sphere S shows its binding energy 290.3 eV and 163.8 eV corresponding to C1s and S spectra respectively, 3 confirmed the S-O bond with green-red sphere shows its binding energy 534.9 eV and 165.0 eV corresponding to O1s and S spectra of GO-ZnS sandwich material (see supplementary information).

Figure 5 reveals the information obtained from XPS spectra of GO-ZnS sandwich material. Here to confirm the hetero nanogate type structure coordinated with Zn through S between two GO layers, a schematic diagram is shown where the variation of the binding energy (B. E.) with the formation of nanogate has been illustrated. The B.E. at 163.7 eV and 165.0 eV signify the C-S and S-O bond in sandwich material respectively^45^. The detailed XPS studies for GO and ZnS has been shown in Section S2.3 (see Supplementary Information).

## Charge Transfer Mechanism

**Figure 6:**
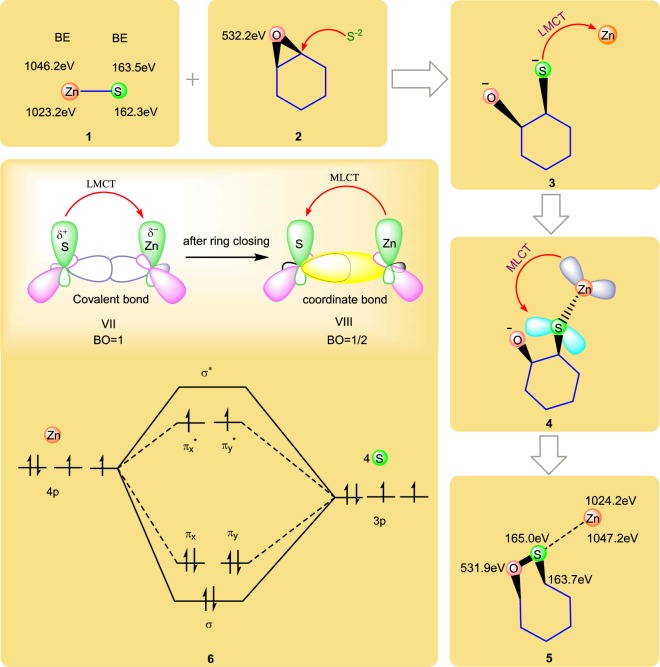
Charge transfer mechanism based on XPS data. (1, 2) shows the B.E. of ZnS; (2) B.E. of epoxide group in GO; charge transfer from (3) S to Zn (LMCT); (4) Zn to S (MLCT); (5) confirmation of charge transfer and formation of hetero nanogate by shifting of B.E. (6) molecular orbital (MO) diagram of Zn coordinated with four S atom. The formation of coordination bond after the ring closing is confirmed by the change in bond order of Zn-S from 1 to 1/2.

To further confirm the charge transfer mechanism and nanogate structure, following strategies has been utilized. For example, in a compound A-B, B.E. of A increases when charge transfer occurs from A to B (Concept 1) as well as when the bond order decreases (Concept 2). It is observed that the B.E. of metal in metal complex is higher than corresponding metal^46^. To consider the above two facts, Fig. 6 exhibits charge transfer mechanism in nanogate structure. Pictorial 1 show the B.E. of Zn and S were (1023.2 eV and 1046.2 eV) and (162.3 eV and 163.5 eV) respectively, whereas B.E. of epoxide group was 532.2 eV (Pictorial 2). During the synthesis of 4 member nanogate type hetero structure, Pictorial 3 shows the covalent bond formation *in-situ* between S and Zn atom (see Mechanism). So, here metal has the tendency to accept the electrons (4p orbital of Zn from 3p orbital of S) thereby giving a partial negatively charged Zn and partial positively charged S via ligand to metal charge transferr (LMCT) (see Pictorial 6 - Mechanism). That is why S is bonded with O atom to form a 4 member nanogate type hetero structure and subsequently Zn transferred the electron in π* orbital of S through metal to ligand charge transferred (MLCT), thereby converting the covalent bond into coordination bond (see Pictorial 4/6 and Mechanism). Pictorial 5 shows the increase in B.E. of Zn (1024.2 eV, 1047.2 eV) indicates that charge transferred Zn to S (Concept 1). B.E. of S also increases that confirms the decrease in bond order (covalent to coordinate) which also proves the Concept 2. According to concept 1, B.E. of Zn should increase when there is a charge transfer to S, and accordingly the B.E. of S should be decrease, but in our proposed nanogate type structure B.E. of S increases because S also shares the electrons to form bond with C and O atom too. So that the B.E. of S was atom observed at 163.7 eV and 165.0 eV corresponding to C-S bond and S-O bond respectively^45^. This is the reason why B.E. of O decreases from 532.2 eV to 531.9 eV.

## Molecular Orbital Diagram

Now the questions arise. What is the bond order between Zn and S atom in sandwich structure? To understand this, we presented a molecular orbital (MO) diagram in Pictorial 6. It indicates the bonding between 4S atom and Zn atom in GO-ZnS sandwich material as Zn coordinated with 4S atom (see Mechanism). Each S atom transferred one electron from 3p orbital to 4p orbital of Zn, so that four S atom transferred 4 electrons in 4p orbital of Zn by LMCT (Pictorial 3) and 3 electrons remains in 3 p orbital of S. Out of these three electrons in S, one electron each is shared with C and O atom to form C-S and S-O bond, respectively thereby leaving one electron in 3p orbital of S atom. So, the remaining one electron each from the four S atom (4 electron in total) are participating in bonding with Zn through MLCT (Pictorial 4) leading to form a coordination bond. The bond order between Zn and S is 1/2 which is less than 1 (covalent bond) further confirms concept 2.

**Figure 7:**
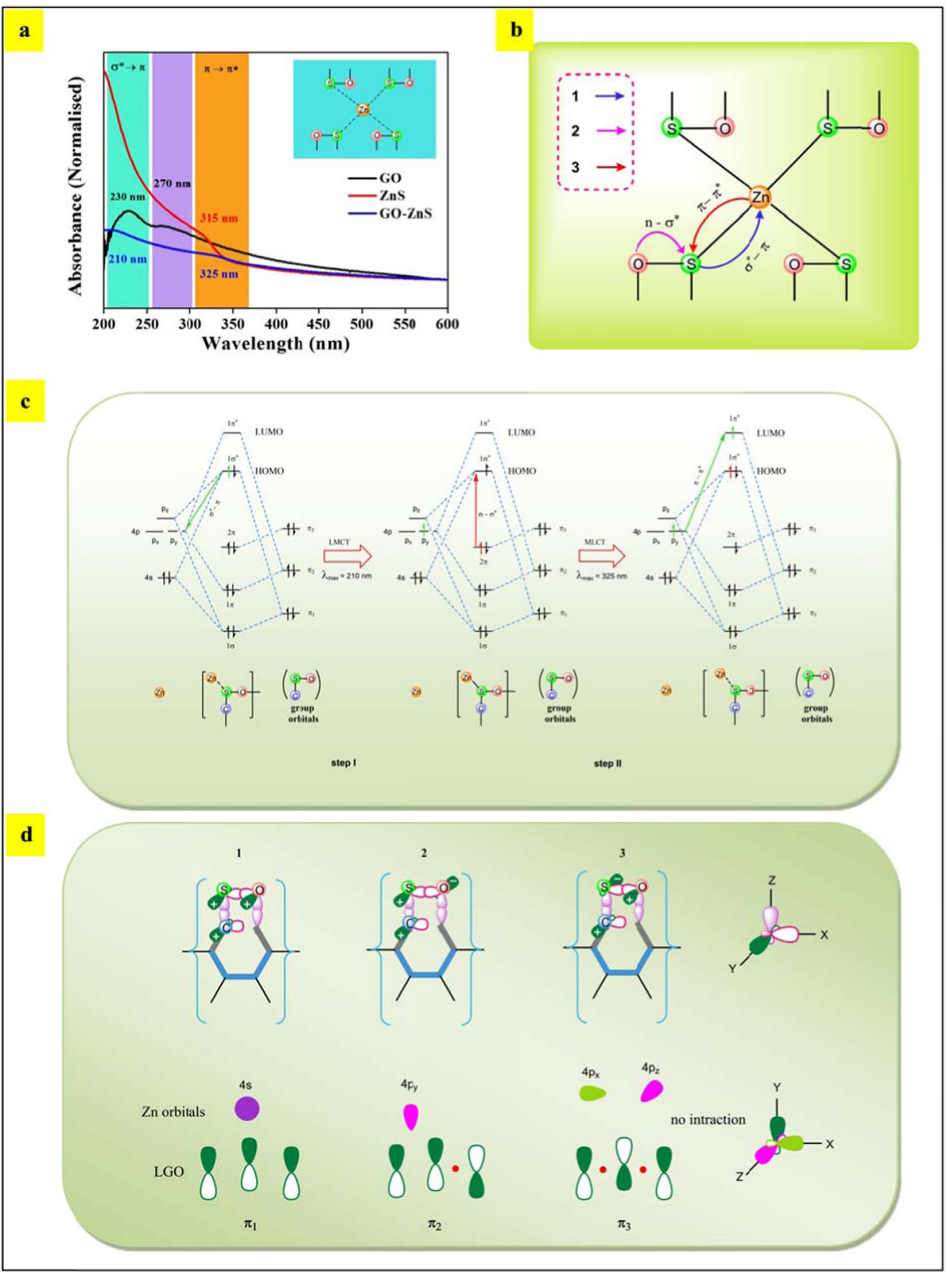
UV-Visible spectra and Molecular orbital diagram representation of ligand group orbitals and Zn orbitals, (**a**) UV-Visible spectra of GO, ZnS and GO-ZnS sandwich structure, inset: coordinate bond formation in GO-ZnS (**b**) sequence of the possible electronic transition in GO-ZnS (**c**) confirmation of observed transition in sandwich nanogates structure with the help of various MO diagram mechanism. The assignment of transition in UV-Visible spectra as well as confirmation of bond order between zinc and sulphur atom, step I shows the blue shift (210 nm ← 230 nm) and step II shows the red shift (315 nm → 325 nm) through the transition π ← σ* and π → π* with confirmed the LMCT and MLCT respectively. (**d**) formation of ligand group orbitals (C-S-O), positive and negative sign indicate the orbital phase in same direction and opposite direction respectively and below one shows their interaction with metal orbitals.

Inorder to have a better and clear chemistry behind the charge transfer process and nanogate formation, it is necessary to consider the group orbital of (C-S-O) as C-S and S-O bonds are also having an impact to the overall nanogate formation. Here, we have explained through the MO diagram between the Zn and the group orbital of (C-S-O) (see Fig. 7). From the UV spectra of GO-ZnS (Fig. 7a), we observed two shift of peaks viz blue shift (210 nm ← 230 nm) that is π Zn(4p) ← σ* which strengthen the C-S bond while the red shift (315 nm → 325 nm) that is π Zn(4p) → π* which weakens the C-S bond^47^. The red shift also weakens the Zn-S bond due to the back electron donation thereby decreasing the bond order between Zn and S leading to the formation of a coordination bond (as shown in inset of Fig. 7a). The sequence of the electron transition is shown in Fig. 7b where initially S donates an electron to Zn subsequently followed by the ring closing (bond between S-O) and finally the back transfer from Zn to S. The above point can be clearly explained by considering the MO diagram presented in Fig. 7c. Here. a three MO diagram is drawn to explain the transition through a two-step mechanism in GO-ZnS sandwich structure^48^. In step I, electron is transferred from 1σ* of group orbital (mainly comes from S atom) to empty 4p_y_ orbitals of Zn (increase the bond ordered between Zn and S) confirming the LMCT at λ_max =_ 210 nm. Now in step II, electron transition occurs from nonbonding (2π) orbital to antibonding**1**σ* orbital, followed by the back donation from Zn 4p_y_ orbital to 1π* through the transition π → π* confirming the MLCT at λ_max =_ 325 nm. Here, the bond order between Zn and S is also found to be 1/2 which is a characteristic of a coordination bond.

The construction of ligand group orbitals between C, S and O atom was done through the frontier molecular orbital (FMO) approach (Fig. 7d). Empty p_y_ orbitals of all three atoms (C, S and O) can form group orbitals through the interaction between lobes of adjacent atoms. Considering the orientation of the lobes in same phase (positive sign lobe) or opposite phase (negative sign lobe), p_y_ orbitals of all three atom can interact in three possible fashion (**1**, **2** and **3)** leading to formation of group orbitals as π_1,_ π_2_ and π_3._ Based on the no of nodes present, the orbitals energies is in the following order; π_1 ˂_ π_2˂_ π_3._ As shown in Fig. 7d the Zn 4s orbital interact with lower-lying π_1_group orbital and form 1σ orbital, then metal 4p_y_ orbital interact with higher-lying π_2_ group orbital and resulting 1π orbital because of their adapted symmetry. But in case of (4p_x_ and 4p_z_) orbitals, due to the mismatch of the phase orientation with the higher-lying π_3_ group orbital, bonding is not possible thereby making π_3_ group orbital as nonbonding.

## Morphological Analysis

**Figure 8:**
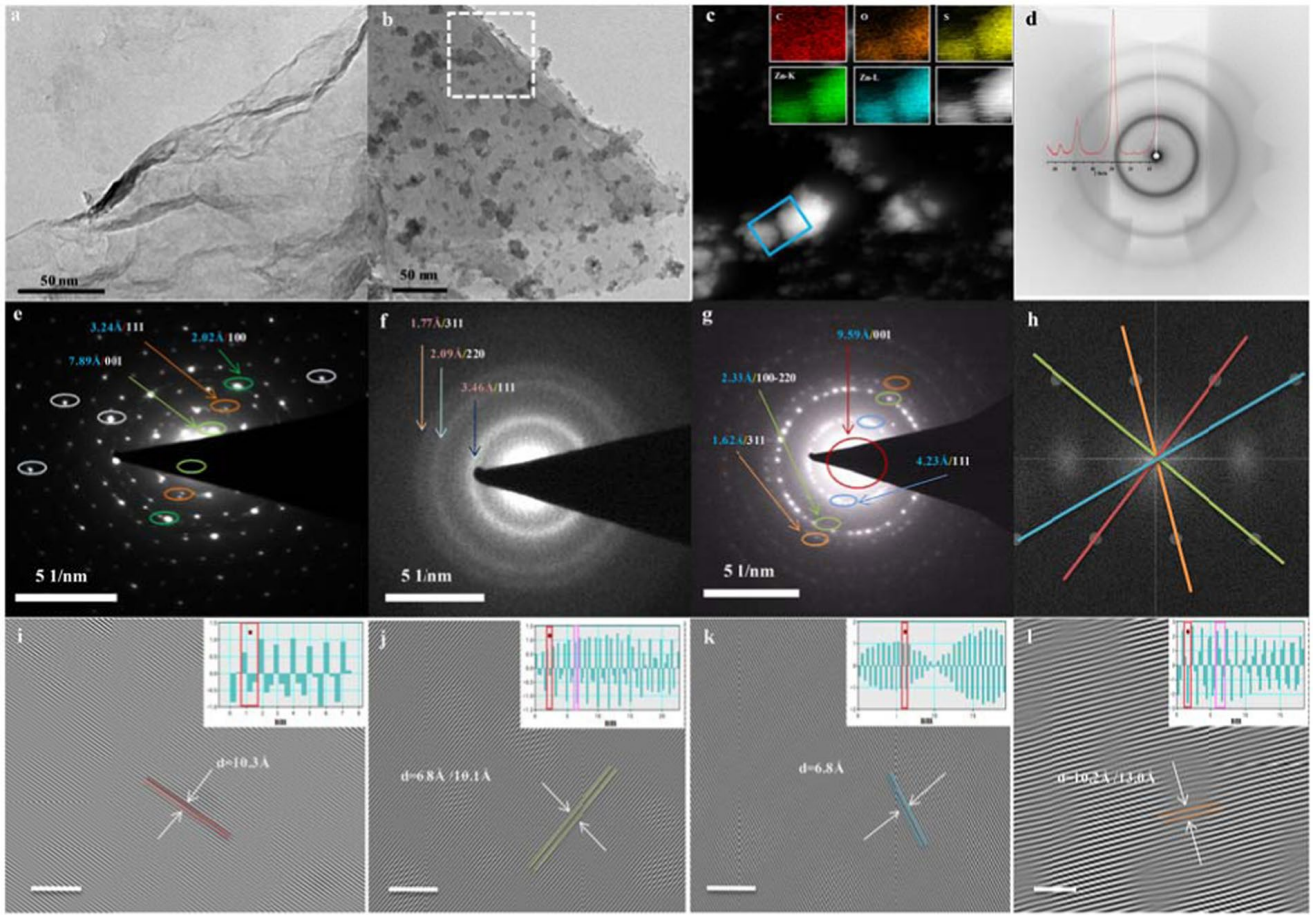
(**a**,**b**) TEM image of GO and GO-ZnS sandwich structure respectively. (**c**) HAADF-STEM and inset their elemental mapping of different atom in GO-ZnS sandwich material. (**d**) 2D image of WXRD of GO-ZnS sandwich material overlapped with WXRD spectra. (**e**–**g**) SAED pattern of GO, ZnS QDts and GO-ZnS sandwich material respectively. (**h**) FFT taken from white dotted area in figure (**b**). (**i**–**l**) Inverse Fast Fourier Transform (IFFT) diffractogram (with scale bar 5 nm) and inset is their plot profile of *d*-spacing of different plane indicated by different colour in corresponding FFT image. (**h**) This shows the variation in d spacing between graphene layers atom intercalated by nanogate structure.

Figure 8a,b shows the TEM images of GO and GO-ZnS where GO forms a perfect sheet like morphology, while in GO-ZnS, there is a successful incorporation of the ZnS moiety in the GO sheets. (c) shows the HAADF-STEM indicates GO-ZnS sandwich material, where the presence of C, O, Zn and S were indicated in the inset of (c). (d) shows the 2D image for the WXRD spectra for the sandwich material where there is a complete matching of the peak and the intensity of the pattern. (e) indicated the SAED pattern of GO in which different colour ring denoted the different plane. 001 plane (observed at 2θ = 11.2in XRD), is the characteristic of GO with d spacing 7.89 *Å*^49^. Other two planes 111 and 100 (observed are at 2θ = 26.6 and 43.1) originated from the graphite (rhombohedral structure with space group *R-3m*) with d spacing of 3.24 *Å* and 2.02 *Å* respectively. (f) shows the SAED pattern of ZnS (cubic structure with space group *F-43m*)where three plane (111, 220 and 311) are observed with their d- spacing of 3.46 *Å*, 2.09 *Å* and 1.77 *Å* respectively. As observed in (g), two planes with similar d-spacing such as 111 plane (GO and ZnS), 100 (GO) and 220 (ZnS) overlap with each other and is observed as a combination of both in the sandwich material. The variation in the d-spacing as calculated from XRD and SAED pattern has been tabulated in Table S3. The (111) plane of GO and ZnS merged together giving a new pattern with d spacing 4.23 *Å* while 100 (GO) and 220(ZnS) emerges with d- spacing of 2.33 *Å*. The important feature of this sandwich material is the characteristic plane (001) with d-spacing of 9.59 *Å* which perfectly matched with XRD calculated value of 9.56 *Å*.

It is well-known that Fast Fourier Transformation (FFT) can be used in a number of applications to predict the exact result^50^. In SAED pattern, due to the huge interference from the source light, (001) plane of GO were not clearly observed. So, FFT diffractogram (h) were analysed from the rectangular dotted area in (b). It shows the different plane with different colour line lines. (i-l) indicted the Inverse - FFT taken from corresponding plane in (h) which denotes their respective d-spacing value (inset shows their corresponding plot profile). Here, the d-spacing of 6.8, 10.1 and 13.1 *Å* were observed which can be assigned to the (001) plane of GO. The variation can be further explained by the schematic diagram (Fig. S5, see Supplementary Information, Section S3) where the GO interlayer distance varies due to the incorporation of the nanogate structure.

Thermogravimatric analysis indicated the formation of sheet and stable structure of GO-ZnS sandwich material (Section S2.4, See Supplementary Information). Detail analysis about the optical properties has been discussed in Supplementary Information (Section S4).

## Computational Study of Graphene Nanogates Structure

### To prove the proposed hypothesis of the nanogates structure, we perform the Density Functional Theory (DFT) calculations

**Figure 9:**
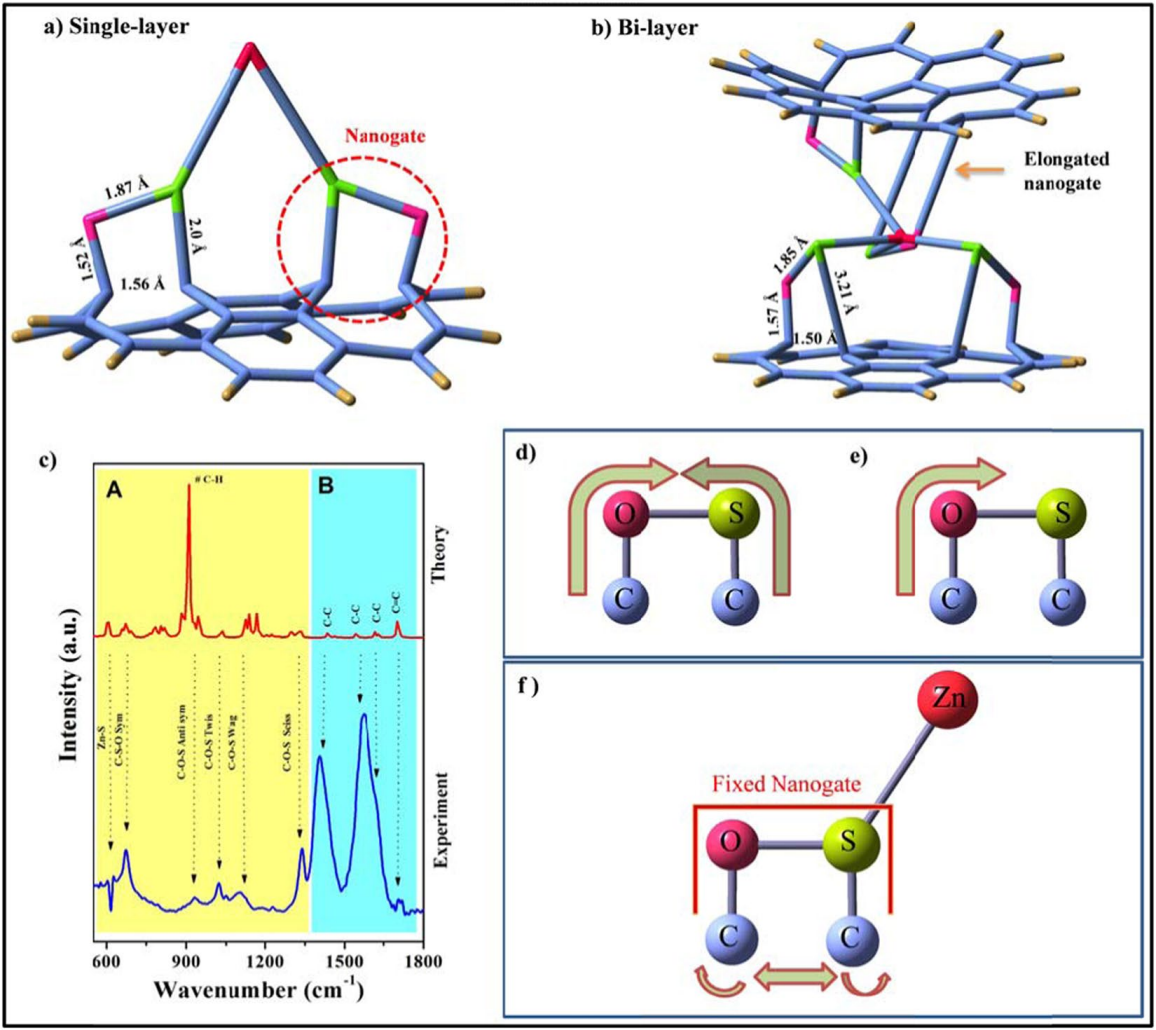
DFT optimized (B3LYP/LANL2DZ) computed nanogates structure on (**a**) single layer of graphene and (**b**) between the bi layers of graphene respectively. (**c**) Infrared (IR) spectra of the nanogates structure calculated from density functional theory (DFT) along with experimental data. Schematic diagram of different modes of vibration (**d**,**e**) C-S-O and C-O-S as well as (**f**) fixed nanogate on interaction with Zn atom (see video).

For optimization, B3LYP functional, 6-31 G basis set and LANL2DZ were used for metal coordination^51^. Figure 9a,b shows the nanogate formation in a single and bilayer GO sheets, respectively.

Here, the observed bond length are as follows C-O (1.52 Å), C-S (2.0 Å) and S-O (1.87 Å), while for C-C (1.56 Å) which is a bit longer as compared with the C-C bond in the normal graphene sheet resulting in the defect formation in the proposed nanogate structure. While in the bilayered GO sheets, there is also a successful optimization of the nanogates with one slightly elongated nanogate structure which might be due to the vander waals repulsion between layers. Still the formation of the nanogate structure indicates the stable formation in-between the layers as observed from the flattening of the GO sheets as compared with the single layer conformation. There is also a negligible variation in the bond length of C-O (1.57 Å), C-S (3.21 Å), S-O (1.85 Å) and C-C (1.50 Å).

Figure 9c shows the IR spectra of DFT calculated spectra along with the experimental data. All the observed vibrations are assigned under two categories, A) “within” the nanogate formation and B) “bonded/adjacent” to the nanogate structure. The vibrations at 670 cm^−1^_,_ 930 cm^−1^, 1022 cm^−1,^ 1120 cm^−1^ and 1337 cm^−1^ were assigned to C-S-O symmetric stretch, C-O-S anti symmetric stretch, C-O-S twisting, wagging and scissoring respectively (See Video S1,S2, Supporting Information). Due to the interaction of Zn with S, the O-S bond become rigid thereby allowing the free movement of the two C atoms in the nanogate structure (Fig. 9f and Video S3, Supporting information). The C=C vibration of the adjacent rings were also observed at 1622/1700 cm^−1^. The intense peak observed in the theoretical spectra at 910 cm^−1^ is due to C-H stretch, which is not observed in the experimental data. In the theoretical assumption, we took a minimum no of atoms for the nanogate formation where all the periphery C atom have C-H bond, while in the experimental results, its is a large GO sheets and the concentration of C-H is quite minimized, so corresponding peak of C-H were not observed in the experimental spectra.

## Summary and Scope

In concise, we have reported the synthesis of new sandwich material with a very simple and novel synthesis method, by which a nanogate structures between two GO layers has been discovered. The formation of the defects in the GO sheets with the incorporation of nanogates has been explained by a step-wise reaction mechanism, charge transfer process, frontier MO theory, theoretical structure elucidation, various experimental analyses and DFT calculation. The role of the spacer (Zn acetate – PEG) in increasing the interlayer distance between the GO sheets. The formation of C-S and S-O bond leading to the nanogate structure formation thereby making defects in the already stable structure of GO. The coordination bond formation of Zn and S thereby holding the two GO sheets. The confirmation of the new bond formation in the nanogate through the charge transfer mechanism.

With this understanding of the nanogate structure in-between the GO sheets, it will open a huge avenue for the untapped part of the graphite (various functional groups in the basal plane) leading to the enhancement in the properties of the composite materials. Due to the nanogate formation, there might be a possibility of enormous generation of electrons on excitation of the composite materials, which can be a boom for various applications like photocatalysis, water splitting, solar cell, etc.

## Supplementary information

C-S-O Vibrational Mode

C-O-S Vibrational Mode

C-C Vibrational Mode

Supplementary Info
