# Supplementary figures and images for: Synthesis of nanogate structure in GO-ZnS sandwich material
Source: Sci Rep. 2019 Jan 30;9:937. doi: 10.1038/s41598-018-37396-8 (PMC6353954; doi:10.1038/s41598-018-37396-8)

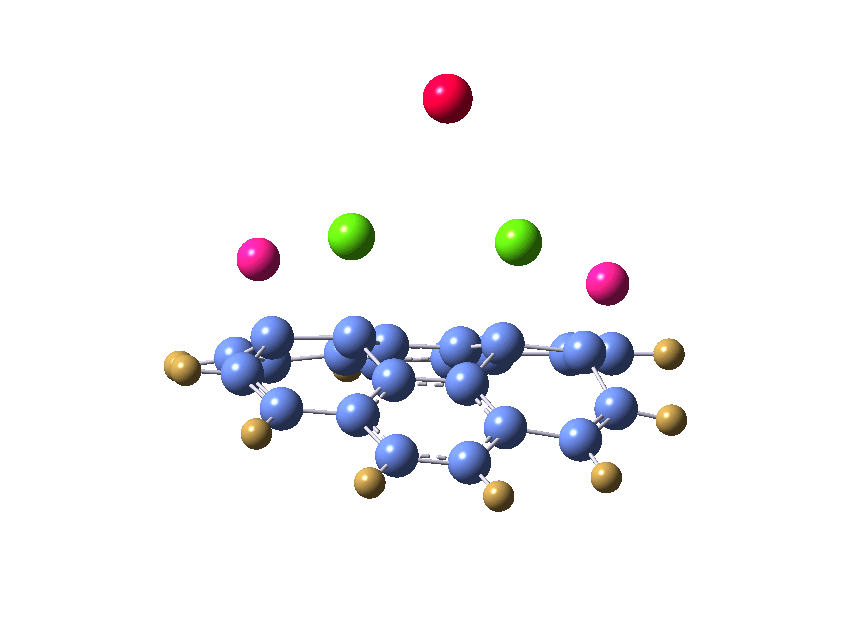

Supplement: Supplementary file 1 — C-S-O Vibrational Mode [file 41598_2018_37396_MOESM1_ESM.gif]

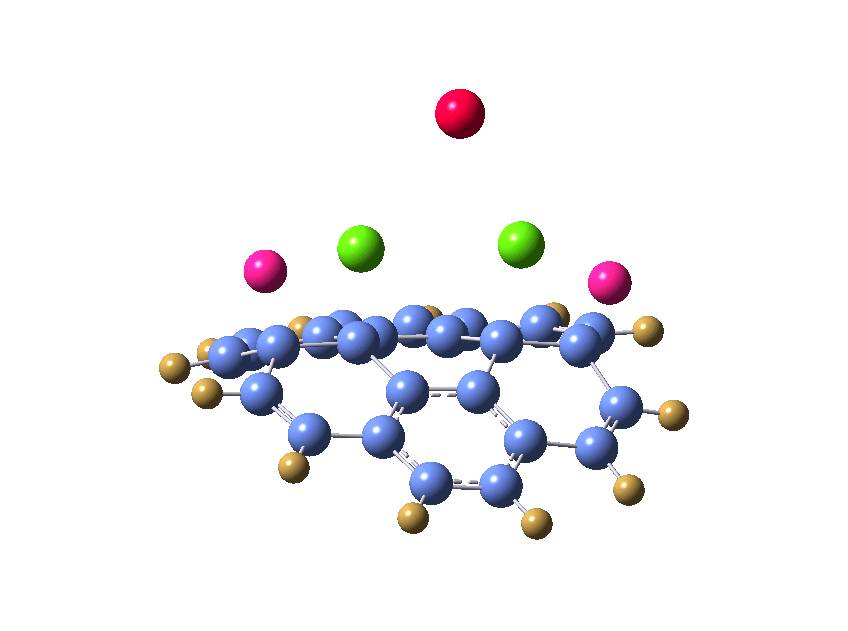

Supplement: Supplementary file 2 — C-O-S Vibrational Mode [file 41598_2018_37396_MOESM2_ESM.gif]

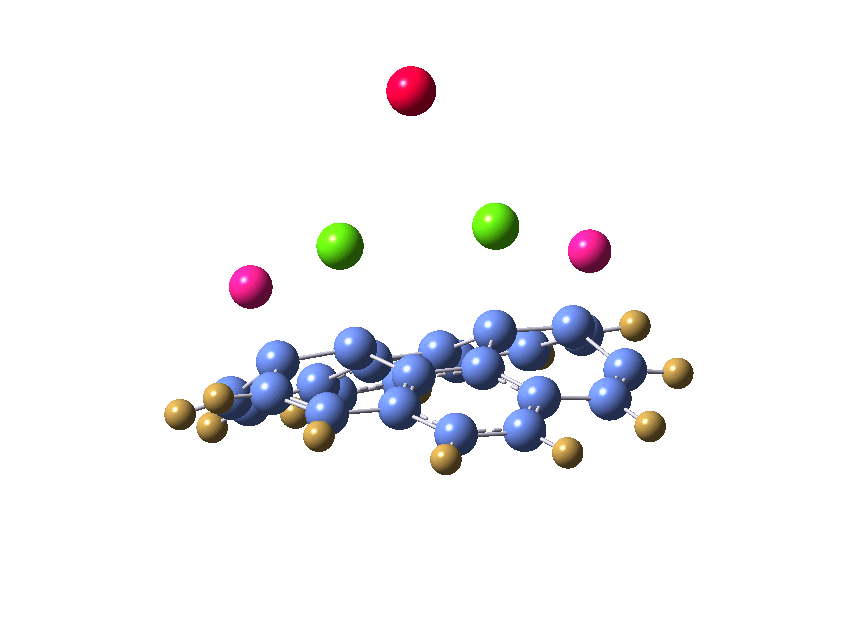

Supplement: Supplementary file 3 — C-C Vibrational Mode [file 41598_2018_37396_MOESM3_ESM.gif]
