# Supplementary material for: Synthesis of nanogate structure in GO-ZnS sandwich material
Source: Sci Rep. 2019 Jan 30;9:937. doi: 10.1038/s41598-018-37396-8 (PMC6353954; doi:10.1038/s41598-018-37396-8)
Supplement: Supplementary file 4 — Supplementary Info [file 41598_2018_37396_MOESM4_ESM.docx]

## Supplementary Information

**Synthesis of nanogate structure in GO-ZnS sandwich material**

Praveen Kumar^1^ and Meitram Niraj Luwang^1,2,^*

*^1^Chemical Engineering and Process Development Division, National Chemical Laboratory, Pune - 411008 (INDIA)*

*^2^Academy of Scientific and Innovative Research (AcSIR), CSIR-Human Resource Development Centre, Campus Postal Staff College Area Ghaziabad 201002 India*

****Corresponding author***

*mn.luwang@ncl.res.in* (Meitram Niraj Luwang)

Ph: (+91) 20 2590 2950

Fax: (+91) 20 2590 2621

**Table of Contents**

**Section 1: Synthesis of new sandwich material and its competitive**

S 1.1: Synthesis and Experimental section……………………………………………..… 3

S 1.2: Characterization techniques…………………………………………………......… 4

**Section 2: Structural based characterization and their study**

S 2.1: Solid State Nuclear Magnetic Resonance (NMR)…………………………….....…5

S 2.2: Powder X ray diffraction (XRD)………………………………………….....……..5

S 2.3: X- ray photoelectron spectroscopy(XPS)………………………………... …..……6

S 2.4: Thermo Gravimetric Analysis (TGA)………………………………...……...……..9

**Section 3: Morphological study**

S 3.1: Transmission electron microscope (TEM)……………………………..…...… …10

S 3.2: Atomic force microscope (AFM)…………………………………………...……..11

**Section 4: Optical analysis**

S 4.1: Ultraviolet/visible spectroscopy………………………………………...…………11

S 4.2: Photoluminescence spectroscopy……………………………………………...…..12

**Section 5: Applications**

S 5.1: Photocatalytic activity………………………………………..……………………13

**Section 6: References and Notes**………………………………………..…………………..14

**Section 1: Synthesis of new sandwich material and its competitive**

***S 1.1: Synthesis and Experimental section***

**Materials and Method:** Graphite powder(99.99%), KMnO_4_(≥99.0%), Zn(CH_3_COO)_2_.2H_2_O(≥98%), Polyethylene Ethylene Glycol (PEG) Bioultra-4000, Na_2_S.xH_2_O(59-65%), were obtained from Sigma-Aldrich and Sulfuric Acid(98% GR), Hydrochloric Acid(35-38% GR), H_2_O_2_(30%) were obtained from Fisher Scientific Chemicals India.

**Synthesis of Graphene oxide Nanosheet:** Graphene Oxide was synthesized by modified Hummer’s method.[^1^](#_ENREF_1) Initially, 2g of graphite powder was mixed with 100 ml concentrated H_2_SO_4_ with continuous stirring. Then, 8 g of KMnO_4_ (who act as an oxidizing agent)was added slowly to the above solution and the temperature was maintained below 5 ^°^C for 2 hr by using ice bath, to neutralized the exothermic nature of the reaction. 100 ml distilled water along with3-4 ml of 30% H_2_O_2_ was added simultaneously to above solution mixture. After the colour of the solution changes from dark black to yellow, the final solution mixture was filtered and washed three or four times with 1M HCl and DI water. Finally, the GO residue was dried in oven at 60 °C for an overnight.

**Synthesis of zinc sulphide quantum dots:** 2.2 g of zinc acetate was added into 20 ml of distilled water followed by 40 ml of poly ethylene glycol (0.5 M) and the solution is maintained with continuous stirring at 80 ^°^C for 10 mins. After that, 20 ml of sodium sulphide solution (0.5M) was added drop wise and the solution was stirred for another 12 hrs. The Zinc Sulphide quantum dots were collected by centrifugation with regular washing 3-4 times with ethanol and water. The collected residue was dried in vacuum oven at 60 °C for an overnight.

**Synthesis of GO-ZnS sandwich material:** In this novel way of synthesis, 0.0219 gm of graphite oxide powder was added to 20 ml of distilled water and the solution is allowed to stirred for 10 mins at 60-70 °C. 20 ml of 0.5 M Zn (CH_3_COO)_2_ solution containing equivalent amount of PEG-4000 (w/w) w.r.t GO was added to the above solution. After continuous stirring for 15 mins. 20ml of Na_2_S solution (0.5M) was added dropwise to the above solution mixture and kept it for another 12 hrs with continuous stirring at 60-70 °C. The solution is transferred into 100 ml Teflon lined stainless steel autoclave at 180 ^0^C for 12 hours. The GO-ZnS sandwich material is obtained with regular washing with acetone to remove the impurities. The collected residue is dried at vacuum oven at 60 °C for an overnight.

***S 1.2: Characterization techniques***

PANalyticalX'pert pro X-ray diffractometer using Cu Kα (λ = 1.542 Å) radiation was used to determine the crystal structure of synthesized materials with operating voltage 40 kV and operating current was 30 mA. Bruker Tensor 27 IR was used for the functional group analysis. For elemental composition and binding energy, X-ray photoelectron spectra (XPS) was recorded with custom built ambient 20 pressure photoelectron spectrometer (APPES) (Prevac, Poland). Optical absorbance was observed by SHIMADZU UV-3600 plus UV-VIS-NIR spectrophotometer and photoluminescence spectra was recorded by PTI Quantum Master 400 spectrophotometer. Raman spectra were measured by RenishawInVia Microscope Raman spectrometer using 532nm excitation..

Solid state 1D NMR spectrum was recorded at an external field Bₒ of 9.38977 T using JEOL 400 spectrometer [400 MHz] operating at a ^13^C Lamor frequency of 100.5253MHz. ^1^H-^13^C Cross-Polarisation Magic Angle Spinning (CPMAS) were measured with 1s recycle time, and 1 ms contact time. The ^13^C chemical shift were calibrated against TMS(δ= 0ppm).Direct ^13^C pulse with MAS operating in dipolar dephasing mode, 10000 scans with a 50 μs dephasing interval time to FID acquisition.

For the morphology analysis, CM-200 transmission electron microscope FEITECNAI 3010electron microscope operating at 300 kV (Cs = 0.6 mm, resolution1.7 Å) were utilized for TEM and HRTEM images. For the TEM measurement, the powder samples were grounded and dispersed in ethanol. A drop of the dispersed particles was put over the carbon-coated copper grid and evaporated to dryness at room temperature. High Angle Annular Dark Field-Scanning Transmission Electron Microscope (HAADF-STEM) and Inverse Fast Fourier Transform Diffractogram (IFFT)were also analysed. Thermogravimetric analysis (TGA) were carried out in a SDT Q600 TGDTA analyser in the temperature range of 25 - 900 °C on under N_2_ atmosphere at a heating rate of 10 ºC min^-1^.

All the AFM measurements were done at room temperature in the tapping mode using a Nanosurf AFM. The samples were prepared over a silica wafer.

**Section 2: Structural based characterization and their study**

***S 2.1: Solid State Nuclear Magnetic Resonance (NMR)***

**Figure S1** shows the ^13^C-SSNMR spectra of synthesize GO where all the peaks are correlated with previously reported values of GO.[^2^](#_ENREF_2) The peaks are at 61ppm, 71 ppm, 101ppm and 131 ppm corroborate to the epoxide, hydroxyl, lactol moiety and sp^2^ cluster respectively while the other minor peak at 168 ppm and 191 ppm corresponds to carbonyl and carboxylic group.

***Figure S1*|** *NMR Spectra,* *^13^C solid state NMR spectra of synthesized graphite oxide where different colour region shows the different functionalities exist in GO.t_cp_= 1ms, 67,000 scans.*

***S 2.2: Powder X ray diffraction (XRD)***

The powder XRD patterns of GO, ZnS, and GO-ZnS sandwich material were shown in **Figure S2**. The XRD spectra of ZnS quantum dots shown in pattern **I** in which three peaks were observed at *2θ*(degree) = 29.2, 48.4 and 57.7 corresponds to 111, 220 and 311 plane respectively (JCPDS No. 05-0566). It has the cubic zinc blend structure with space group *F-43m*. Pattern **II** shows the XRD spectra of GO where the 001,111 and 100 plane of GO were assigned to the peaks observed at *2θ* (degree) = 11.2, 26.6 and 43.1respectively (JCPDS No. 01-075-2078). In GO, graphite layers has rhombohedral unit cell with space group *R-3m*. In pattern **III** (XRD spectra of GO-ZnS), the peak position of 001 plane of GO is shifted towards lower *2θ*values (observed only in WXRD, see main text, Figure X) while there are no observable changes for ZnS peak (**Table S1**).

***Table S1*|** *Comparison of 2θ, FWHM and area under the curve as calculated from W-XRD pattern for GO, ZnS and GO-ZnS sandwich material.*

| Sl. No. | GO | | | ZnS | | | GO-ZnS | | |
| --- | --- | --- | --- | --- | --- | --- | --- | --- | --- |
|  | ***2θ*** | ***FWHM*** | ***Area*** | ***2θ*** | ***FWHM*** | ***Area*** | ***2θ*** | ***FWHM*** | ***Area*** |
| 1 | 11.2 | 3.68 | 3317 | - | - | - | 9.2 | 4.7 | 646 |
| 2 | 26.63 | 1.03 | 128.4 | - | - | - | 18.3 | 0.65 | 1.62 |
| 3 | - | - | - | 29.2 | 2.84 | 222.5 | 29.1 | 2.11 | 408 |
| 4 | 43.1 | 1.5 | 542 | **-** | **-** | **-** | 39.0 | 0.28 | 0.676 |
| 5 | **-** | **-** | **-** | 48.4 | 4.66 | 97.2 | 48.4 | 2.07 | 92.93 |
| 6 | **-** | **-** | **-** | 57.7 | 7.72 | 133.8 | 57.1 | 1.12 | 9.96 |

***Figure S2*|** *X-ray diffraction pattern I,* *shows the XRD pattern ZnS with three plane 111, 220 and 311.* ***Pattern II*** *corresponding to GO shows the three planes 001,111 and 100, plane 001 is the characteristic feature of GO and remaining two planes comes from the graphite.* ***Pattern III****, shows the GO- ZnS sandwich material.*

***S 2.3: X- ray photoelectron spectroscopy (XPS)***

X-ray photoelectron spectroscopy (XPS) is used to identify the elemental composition and functional group bonded with different atom in a compound. **Figure S3** shows the XPS spectra of GO-ZnS sandwich material along with GO and ZnS spectra. **(a)** and**(b)** shows the deconvoluted C1s and O1s curve of GO respectively. In **(a),** five peaks at 284.1eV, 285.3eV, 286.6eV, 288.1eV and 289.7eV corroborate the functional group sp^2^-sp^3^ hybrid cluster, hydroxyl(-C-OH), epoxy(C-O-C), carbonyl(-C=O) and carboxylic(-COOH) group respectively. **(b)** exhibit two peaks at 532.2eV and 533.6eV corresponding to epoxy and hydroxyl group respectively.[^3^](#_ENREF_3)^,^[^4^](#_ENREF_4)**(c)** and **(d)** indicate the new peaks observed in GO-ZnS sandwich material corresponding to C1s and O1s spectra respectively.

***Figure S3*|** *X-ray photoelectron spectroscopy* *of* ***(a)*** *C1s spectra of GO,* ***(b)*** *O1s spectra of GO,* ***(c)*** *C1s spectra of GO-ZnS sandwich material,* ***(d)****O1s spectra of GO-ZnS sandwich material,* ***(e)****Zn 2p peaks in ZnS,* ***(f)****S 2p peaks in ZnS,****(g)*** *Zn peaks in GO-ZnS sandwich material and* ***(h)****S peaks in GO-ZnS sandwich material.*

In C1s spectra, peak at 284.8eV corresponding to sp^2^ hybrid cluster originate from GO layers. Other peaks were observed at 287.3eV and 290.3eV indicate the oxygen attach with sp^2^ carbon atom and C-S bond formation respectively and lead to construct a four member hetero nanogate structure. From O1s spectra, the two peaks were observed at 531.9eV and 534.9eV consistent with S-O bond and oxygen atom attach with sp^2^ carbon atom on graphene layer respectively. **(e) and (f)**shows the peaks of Zn and S in ZnS quantum dots. From **(e),** peaks at 1046.2eV and 1023.2eV denote the zinc 2p state (1/2 and 3/2 respectively). From **(f)** peaks were observed at 163.5eV and 162.3eV indicate the S 2p state (1/2 and 3/2 respectively).[^5^](#_ENREF_5) **(g) and (h)** remark the new peaks observed in GO-ZnS sandwich material that confirms the formation of hetero nanogate structure sandwich between two graphite layers (G_1_ and G_2_). In **(g)** Zn peaks shifted towards higher binding energy (1047.2eV and 1024.2eV respectively) that clearly shows that zinc form the different bonding arrangement in this new sandwich material (**detail analysis in main text**). From **(h)** two peaks of S observed at 163.7eV and 165.0eV corresponds to C-S bond and S-O bond respectively.[^6^](#_ENREF_6) Andy at el.[^7^](#_ENREF_7) has studied a no. of S-metal containing complexes and observed that higher binding energy of S depends on the changes of environment of the nucleus, radical alternation and complexes charge distribution.[^8^](#_ENREF_8)^,^[^9^](#_ENREF_9) The B.E. of S in metal complexes doesn’t shift more than 2eV[^10^](#_ENREF_10) which also satisfies with our results (**Table S2**).

***Table S2*|** *Comparison of binding energies, FWHM, area under the curve and atomic percentage as calculated from XPS spectra for different elements present in GO, ZnS and GO-ZnS sandwich material.*

| Sl. No. | Elements | GO | | | | ZnS | | | | GO-ZnS | | | |
| --- | --- | --- | --- | --- | --- | --- | --- | --- | --- | --- | --- | --- | --- |
|  |  | ***BE***  ***(eV)*** | ***FWHM***  ***(eV)*** | ***Area***  ***(x 10^-4^)*** | ***Atomic***  ***%*** | ***BE***  ***(eV)*** | ***FWHM***  ***(eV)*** | ***Area (x10^-4^)*** | ***Atomic***  ***%*** | ***BE***  ***(eV)*** | ***FWHM***  ***(eV)*** | ***Area (x10^-4^)*** | ***Atomic***  ***%*** |
| 1 | C1s | 284.1 | 0.88 | 2.1 | 18.30 | - | - | - | - | 284.8 | 1.0 | 0.4 | 8.36 |
| 2 |  | 285.3 | 1.23 | 3.7 | 31.86 | - | - | - | - | 287.3 | 2.0 | 4.4 | 82.36 |
| 3 |  | 286.6 | 1.22 | 2.9 | 25.52 | - | - | - | - | 290.3 | 1.0 | 0.5 | 9.26 |
| 4 |  | 288.1 | 1.3 | 2.4 | 20.59 | - | - | - | - | - | - | - | - |
| 5 |  | 289.7 | 1 | 0.4 | 3.70 | - | - | - | - | - | - | - | - |
| 6 | O1s | 532.2 | 1.84 | 7.8 | 53.16 | - | - | - | - | 531.9 | 0.71 | 0.4 | 10.37 |
| 7 |  | 533.6 | 2.10 | 6.8 | 46.83 | - | - | - | - | 534.9 | 1.6 | 3.8 | 89.62 |
| 8 | Zn | - | - | - | - | 1046.2 | 2.02 | 68.9 | 34.40 | 1047.2 | 1.70 | 85.0 | 32.67 |
| 9 |  | - | - | - | - | 1023.2 | 1.81 | 130.9 | 65.60 | 1024.2 | 1.59 | 175.1 | 67.32 |
| 10 | S | - | - | - | - | 162.3 | 1.6 | 13.0 | 70.03 | 163.7 | 1.5 | 16.8 | 62.78 |
| 11 |  | - | - | - | - | 163.5 | 1.3 | 5.5 | 29.70 | 165.0 | 1.6 | 10.0 | 37.21 |

***S 2.4: Thermo Gravimetric Analysis (TGA)***

**Figure S4** shows the TGA curve for the GO, ZnS and GO-ZnS sandwich materials. There is huge weight loss of around 20% in the temperature range of 100-120 ºC which can be assigned to the loss of loosely bounded water molecule, the weight loss of 20-25% at below 200ºC can be assigned to the loss of carbonyl and carboxylic group in form of CO and CO_2_. The loss of epoxy group and OH group can be assigned to the weight loss of 15-20% at 200-250 ºC. From 300 ºC onwards there is a gradual decrease in the weight loss indicating the formation of rGO^[11](#_ENREF_11)^. For ZnS there is sudden weight loss of 10% at 400 ºC which is due to the oxidation of zinc sulphide[^12^](#_ENREF_12). In the GO-ZnS sandwich material, the weight loss at 400 ºC is due to the ZnS moiety. Form this analysis, it is assumed that due to the incorporation of ZnS moiety in GO, the GO-ZnS sandwich material become more stable due to the formation of the nanogate structure thereby preventing the loss of the functional moiety of GO (epoxy, OH, etc.).

***Figure S4*|** *Thermo gravimetric analysis of* *GO, ZnS and GO- ZnS sandwich material respectively, in the temperature range of 25 - 900 °C on under N2 atmosphere at a heating rate of 10 ºC min-1.*

**Section 3: Morphological study**

***S 3.1: Transmission electron microscope (TEM)***

| Sl. No. | GO | | ZnS | | GO-ZnS | |
| --- | --- | --- | --- | --- | --- | --- |
|  | ***XRD*** | ***TEM*** | ***XRD*** | ***TEM*** | ***XRD*** | ***TEM*** |
| 1 | 7.89 | 7.89 | **-** | **-** | 9.56 | 9.59 |
| 2 | 3.34 | 3.24 | **-** | **-** | 4.83 | 4.23 |
| 3 | 2.08 | 2.02 | **-** | **-** | 2.31 | 2.33 |
| 4 | **-** | **-** | 3.05 | 3.46 | 3.06 | **-** |
| 5 | **-** | **-** | 1.87 | 2.09 | 1.87 | **-** |
| 6 | **-** | **-** | 1.60 | 1.77 | 1.61 | 1.51 |
|  |  |  |  |  |  |  |

***Table S3*|** *Comparison for the d-spacing (Å) as calculated from XRD data and SAED patterns (from TEM images) for GO, ZnS and GO-ZnS sandwich material.*

**Figure S5|** *(a-b) GO layers and GO-ZnS. (c-d) Atomic layers of GO and GO-ZnS. The interlayer distance of GO is 7.8 Å while in GO-ZnS, it shows three different values (6.8, 10.2 and 13 Å). Due to the incorporation of nanogate structure between the graphene layers, the interlayer spacing changes giving a wavy like morphology. This is also well supported by the IFFT diffractogram analysis. The observance of 13Å might be due to the presence of shifting of some adjacent C atoms near the nanogate moiety. (Violet colour sphere indicate the carbon atom of graphene layers and red is the nanogate moiety)*

***S 3.2: Atomic force microscope (AFM)***

**Figure S6,** shows the Atomic Force Microscope (AFM) image and corresponding height profile diagram respectively. Height profile diagram shows the peaks height about 2.4nm that indicates multilayer’s are assemble on each other in GO-ZnS sandwich material. As the calculated distance between two GO layers linked through the nanogates is 9.56*Å*, it shows that the observed peak height of 2.4 nm (24 *Å)* is due to three consecutive GO layers (19.12 *Å*).

**Figure S6|** *AFM image and corresponding height profile diagram of GO-ZnS.*

**Section 4: Optical analysis**

***S 4.1: Ultraviolet/visible spectroscopy***

Ultraviolet/visible spectroscopy was done to study the optical absorbance of GO, ZnS and GO-ZnS sandwich material (**Figure 7a-see main text)**. The optical absorption peak of GO sheets **(a)** was observed at 230 nm (chromophoric group attach with edge side and basal plane of GO sheet) with a shoulder at 270 nm corresponding tosp^2^ and sp^3^ cluster. ZnS quantum dots gave an absorption peak at 320 nm as shown in **(b)**.While in case of GO-ZnS sandwich material**(c)**, absorbance peaks were observed at 210 nm and 325 nm with decrease in absorbance shows that electron transferred from zinc through sulphur to GO layers.[^13^](#_ENREF_13)

In comparison to GO, GO-ZnS sandwich material shown shifting of absorbance peak 230 nm to 210 nm and 270 nm to 325 nm were called blue shift and red shift respectively. The hypsochromic shift (lower λmax=210 nm) indicate that some of chromophoric group were eliminate or reduce from GO and bathochromic shift (longer λmax=325 nm) shows the increase the conjugation in this sandwich material, which represent the electron acceptor and donor nature of chromophoric group present in GO-ZnS sandwich material. That means in composite electron accepted by GO and donor is ZnS. Fatemeh at el.[^14^](#_ENREF_14) recently reported the UV/ Visible spectra of GO-Chitosan composite material and got two peak 210nm and 280nm but the transition are not assigned.

***S 4.2: Photoluminescence spectroscopy***

**Figure S7** shows the photoluminescence spectra of GO, ZnS and GO-ZnS sandwich material excited at 330 nm and spectra were taken between 350 nm to 650 nm wavelengths. There is no observable emission from GO while ZnS quantum dots shows a broad emission in visible region of the spectrum 370 nm to 570 nm with three different peaks at 446(A) nm, 513(B) nm and 557 nm (C). The far edge emission peak at 514 nm which corresponding to recombination of free excitons of ZnS quantum and other emission peak were due to the surface defects and vacancies in ZnS quantum dots. The more number of ZnS atoms exist in ZnS QDts give to the information of an acceptor level within the ZnS optical band gap so that the defect band at 570 nm assign to the transition of zinc interstitial donor to the valence band.

***Figure S7*|** *Figure shows the PL spectra of GO, ZnS quantum dots and GO-ZnS sandwich material. It is clear that quenching is dominant at large extent in GO-ZnS sandwich material.*

The near base base peak at 558 nm assign to the recombination of an electron confine in sulphur vacancy with a hole in valence band of ZnS. In the spectra of GO-ZnS sandwich material, the emission was similar to pure ZnS quantum dots but with slight change in peak position and remarkable quenching of the photoluminescence intensity. The shifting of the peaks in the sandwich materials is due to the new chemical bond formation while the quenching of the emission intensity is due to the absorbance of the excited electron from the ZnS moiety by GO through the nanogates and further participate in the conjugation of GO.

**Section 5: Applications**

***S 5.1: Photocatalytic activity***

From the application point of view, the photocatalytic properties of the synthesised materials were studied against Alizarin-Yellow dye. It is a carcinogenic and mutagenic and main contamination of waste water come out from the textile, leather industry and paints manufacturing.

***Figure S8*|** *Absorbance vs wavelength (A vs W) plot of* ***(a)*** *GO,* ***(b)*** *ZnS and* ***(c)****GO-ZnS sandwich material respectively at different time interval.* ***(d)****Relative Intensity vs Time profile and* ***(e)*** *photographic images of the degradation of the dye after 120 min.*

**Figure S8a-c** shows the catalytic performance of GO, ZnS and GO-ZnS sandwich material at different time intervals (0 min-dark, 20 min, 40 min, 60 min, 80 min, 100 min and 120 min) under UV light. In all the three cases, there is a gradual decrease in the absorbance intensity with the increase in the time. As observed in **(d)**, the photocatalytic behaviour of the GO-ZnS sandwich materials supersede both GO and ZnS. The transfer of excited electron form ZnS to GO sheets (as discussed in absorption and photoluminescence studies) provides the reason for the enhanced photocatalytic activities. The pictorial image for the degradation of the dye by GO, ZnS and GO-ZnS sandwich materials is shown in **(e)** where there is a clear observation of the superior photocatalytic behaviour of the GO-ZnS sandwich materials over GO and ZnS.

**Section 6: References and Notes**

1 Li, Y. & Wu, Y. Coassembly of graphene oxide and nanowires for large-area nanowire alignment. *Journal of the American Chemical Society* **131**, 5851-5857 (2009).

2 Gao, W., Alemany, L. B., Ci, L. & Ajayan, P. M. New insights into the structure and reduction of graphite oxide. *Nature chemistry* **1**, 403-408 (2009).

3 Yan, J. *et al.* Template-assisted low temperature synthesis of functionalized graphene for ultrahigh volumetric performance supercapacitors. *ACS nano* **8**, 4720-4729 (2014).

4 Hsiao, M.-C. *et al.* Thermally conductive and electrically insulating epoxy nanocomposites with thermally reduced graphene oxide–silica hybrid nanosheets. *Nanoscale* **5**, 5863-5871 (2013).

5 Srinivasan, V., Stiefel, E., Elsberry, A. & Walton, R. X-ray photoelectron spectra of inorganic molecules. 21. Sulfur 2p chemical shifts associated with the binding of thiol and thioether groups of transition metal ions. *Journal of the American Chemical Society* **101**, 2611-2614 (1979).

6 Park, M.-S. *et al.* One-step synthesis of a sulfur-impregnated graphene cathode for lithium–sulfur batteries. *Physical Chemistry Chemical Physics* **14**, 6796-6804 (2012).

7 HweeáAw, B., KheongáLooh, K., Hardy, S., LeeáTan, K. & AndyáHor, T. X-Ray photoelectron spectroscopic characterization of [{Pt (PPh 3) 2 (Á 3-S)} 2 PtCl 2],[{Pt 2 (PPh 3) 4 (Á 3-S) 2 Cu} 2 (Á-dppf)][PF 6] 2 [dppf= Fe (C 5 H 4 PPh 2) 2] and other heterometallic aggregates derived from [{Pt (PPh 3) 2 (Á-S)} 2]. *Journal of the Chemical Society, Dalton Transactions*, 3177-3182 (1994).

8 Chatt, J. & Leigh, G. J. The Distribution of Charge in Complex Compounds. *Angewandte Chemie International Edition in English* **17**, 400-407 (1978).

9 Chan, H. S., Hor, T. A., Phang, L.-T. & Tan, K. L. Substituted metal carbonyls: XVI. X-ray photoelectron spectroscopic differentiation of chemically distinct phosphorus environments in unidentate complexes of 1, 1′-bis (diphenylphosphino) ferrocene. *Journal of organometallic chemistry* **407**, 353-357 (1991).

10 Best, S. A. *et al.* X-ray photoelectron spectra of inorganic molecules. 18. Observations on sulfur 2p binding energies in transition metal complexes of sulfur-containing ligands. *Inorganic Chemistry* **16**, 1976-1979 (1977).

11 Yang, H. *et al.* Covalent functionalization of polydisperse chemically-converted graphene sheets with amine-terminated ionic liquid. *Chemical Communications*, 3880-3882 (2009).

12 Theivasanthi, T., Kartheeswari, N. & Alagar, M. Chemical Precipitation Synthesis of Ferric Chloride Doped Zinc Sulphide Nanoparticles and Their Characterization Studies. *arXiv preprint arXiv:1303.2531* (2013).

13 Lightcap, I. V., Kosel, T. H. & Kamat, P. V. Anchoring semiconductor and metal nanoparticles on a two-dimensional catalyst mat. Storing and shuttling electrons with reduced graphene oxide. *Nano Letters* **10**, 577-583 (2010).

14 Emadi, F., Amini, A., Gholami, A. & Ghasemi, Y. Functionalized Graphene Oxide with Chitosan for Protein Nanocarriers to Protect against Enzymatic Cleavage and Retain Collagenase Activity. *Scientific Reports* **7** (2017).
